# Knowledge, attitudes and practices on influenza vaccination during pregnancy in Quito, Ecuador

**DOI:** 10.1186/s12889-020-10061-4

**Published:** 2021-01-07

**Authors:** Carlos E. Erazo, Carlos V. Erazo, Mario J. Grijalva, Ana L. Moncayo

**Affiliations:** 1 Centro de Investigación para la Salud en América Latina (CISeAL), Escuela de Ciencias Biológicas, Facultad de Ciencias Exactas y Naturales, Pontificia Universidad Católica del Ecuador, Apartado, 1701-2184 Quito, Ecuador; 2 Facultad de Medicina, Pontificia Universidad Católica del Ecuador, Apartado, 1701-2184 Quito, Ecuador; 3 Department of Biomedical Sciences, Infectious and Tropical Disease Institute, Heritage College of Osteopathic Medicine, Ohio University, Athens, OH 45701 USA

**Keywords:** Influenza, Influenza vaccination, Pregnant women, Health providers, Ecuador

## Abstract

**Background:**

Vaccination is the most effective way to prevent infection and severe outcomes caused by influenza viruses in pregnant women and their children. In Ecuador, the coverage of seasonal influenza vaccination in pregnant women is low. The aim of this study was to assess the knowledge, attitudes, and practices (KAP) of pregnant women toward influenza vaccination in Quito-Ecuador.

**Methods:**

A cross-sectional study enrolled 842 women who delivered at three main public gynecological-obstetric units of the Metropolitan District of Quito. A questionnaire regarding demographics, antenatal care, risk conditions and knowledge, attitudes and practices related to influenza vaccination was administered. We examined factors associated with vaccination using log-binomial regression models.

**Results:**

A low vaccination rate (36.6%) against influenza was observed among pregnant women. The factors associated with vaccination included the recommendations from health providers (adjusted PR: 15.84; CI 95% 9.62–26.10), belief in the safety of the influenza vaccine (adjusted PR: 1.53; CI 95% 1.03–2.37) and antenatal care (adjusted PR: 1.21; CI 95% 1.01–1.47). The most common reasons for not vaccinating included the lack of recommendation from health care providers (73.9%) and lack of access to vaccine (9.0%).

**Conclusions:**

Health educational programs aimed at pregnant women and antenatal care providers have the most potential to increase influenza vaccination rates. Further studies are needed to understand the barriers of health care providers regarding influenza vaccination in Ecuador.

**Supplementary Information:**

The online version contains supplementary material available at 10.1186/s12889-020-10061-4.

## Background

Pregnant women and infants under 6 months are among the population subgroups considered to be at high risk for serious influenza-related morbidity and mortality, as illustrated during the 1918 and 2009–2010 influenza A (H1N1) pandemics [1]. The mechanical, hormonal, and relevant immunologic alterations that occur during pregnancy may enhance the susceptibility to viral infections and the risk of influenza complications [2, 3]. In 2018, a study estimated that a large proportion of influenza-virus-associated acute lower respiratory infections (ALRI) hospitalizations and in-hospital deaths occurred among young infants and among children in low-income and lower middle-income countries [4]. Influenza can cause primary infections or is also associated with higher rates of secondary bacterial infections [5]. Therefore, maternal influenza immunization could play a role in reducing the burden of all-cause ALRI [4].

In 2012, World Health Organization (WHO) recommended that countries should consider pregnant women as a priority group for vaccination [6]. Several studies have shown that maternal influenza immunization could protect pregnant women from severe complications related to influenza virus infection [7–9], and that infants up to 6 months of age from vaccinated women may also benefit [10–13]. For example, in a pooled analysis of three maternal influenza immunization trials in South Africa, Mali and Nepal, there was 20% reduction in all-cause severe clinical pneumonia in infants under 6 months [10]. In addition, a recent systematic review and meta-analysis reported that maternal influenza vaccination was associated with a 48 and 72% reduced risk of infants having laboratory-confirmed influenza infection and associated hospitalization, respectively [14].

The Ministry of Public Health (MOPH) of Ecuador incorporated the seasonal influenza vaccine to its national vaccination schedule in 2006 and priority groups were included progressively according to WHO recommendations [15]. The MOPH supplies the Northern Hemisphere influenza vaccine free of charge through annual campaigns. In addition, the vaccine must be recommended and offered by health providers, especially those in primary care, during the flu season campaign period. In May 2016, an additional vaccination campaign focusing on priority groups was developed due to an outbreak presented earlier that month [15]. The importance of annual influenza vaccination is highlighted in different media and healthcare centers as well as on MOPH’s website. Despite these efforts, Ecuador has reported low coverage rates of the influenza vaccine in pregnant women (55% in 2015, 63% in 2016, 55% in 2017 and 67% in 2018) [15, 16].

Many studies have tried to determine the factors influencing coverage of vaccination against influenza during pregnancy. Different authors have highlighted that vaccination recommendation by health professionals is the main reason why pregnant women chose to be vaccinated against influenza [17–21]. Other studies have identified additional influences such as: socio-economic characteristics, fear of side effects, doubts about the safety and effectiveness of the vaccine, fear of needles/pain or under-estimation of personal risk [22–25].

Currently, in Ecuador, there is no available data on the factors affecting vaccination among pregnant women. A KAP survey is usually conducted to identify needs, problems and barriers to help plan and implement public health interventions, set program priorities and make program decisions [26]. Therefore, this study aimed to assess knowledge, attitudes, and practices (KAP) of pregnant women regarding influenza vaccination in Quito, Ecuador and to determine the influencing factors associated with vaccination during 2015–2016 campaign. The results of this study may help health authorities plan and implement policies to improve influenza vaccination coverage among pregnant women.

## Methods

### Study design and setting

In Ecuador, two influenza vaccination campaigns were carried out for all priority groups, including pregnant women (December to February 2015–2016 and May 2016). We carried a cross-sectional survey on the knowledge, attitudes, and practices regarding the influenza vaccination during pregnancy from September 2016 to January 2017 in three public hospitals in Quito, the capital of Ecuador. Quito sits at an altitude of 2850 m above sea level and has 2,239,191 inhabitants being the second most populous city in Ecuador [27].

### Study population and sampling

The three public hospitals chosen (Hospital Luz Elena Arismendi, Hospital Isidro Ayora and Hospital Pablo Arturo Suárez) had the highest number of births in 2015 and each hospital is located in a specific area of the city (south, center and north, respectively). In these hospitals, women in immediate postpartum period between 18 and 50 years old were recruited. We interviewed a sample of 854 women (Luz Elena Arismendi, *n* = 168; Isidro Ayora, *n* = 536 and Pablo Arturo Suárez, *n* = 150 women) with probability of selection proportional to the number of live births reported for each health care facility in 2015. This sample size provided 80% power to detect a 10% difference in survey responses to questions about knowledge, attitudes and practices between vaccinated and unvaccinated women (assuming 50% of surveyed women are vaccinated, a 10% non-response rate and alpha = 0.05). Women who did not reside within the Metropolitan District of Quito were excluded from the study.

### Data collection and recruitment

Participant enrollment into the study was carried out by convenience sampling in the postpartum wards of the three hospitals until sample size was reached. Signed informed consent was obtained from each eligible woman interested in enrolling prior to administration of the survey. Illiterate mothers consented by their thumb print after verbal consent. A KAP questionnaire was applied in Spanish by two experienced survey interviewers without medical background who were trained by the authors of the present study (Additional file 1). The survey included questions on demographics, educational level, employment, antenatal care, high-risk conditions, knowledge (influenza, influenza vaccine and severity of influenza), attitudes (perception of vaccine safety and effectiveness) and practices (uptake of influenza vaccine) about influenza vaccine, influenza vaccine during pregnancy, reasons for not receiving vaccination, health provider recommendation and offer of the vaccine. To validate the questionnaire, a team of experts (Influenza Division, CDC, Atlanta, USA) reviewed the items to ensure clarity and adequacy of comprehension prior to administration. Field validation was then carried out and the survey instrument was adjusted accordingly. Self-reported data about influenza vaccination was corroborated through vaccination cards and medical records.

### Statistical analysis

For the analysis of the data, the vaccination report of the mother was used. Age was categorized in four groups: 18–24, 25–30, 31–35, ≥36. Patients were classified as high obstetric risk if they reported having diagnosis of bronchitis, asthma, chronic obstructive pulmonary disease, cystic fibrosis, diabetes, HIV, cardiovascular disease, chronic kidney disease or stroke. We calculated the percentage of women that were vaccinated against influenza by provider recommendation and the offer of influenza vaccination. Among unvaccinated persons, we categorized the main reasons reported for not being vaccinated into 4 main groups: access issues, not wanting or needing the vaccine, concern with safety, lack of offer / recommendation of the vaccine. We also assessed the categorized main reported reasons for not being vaccinated by demographic characteristics, education, number of children, antenatal care, and high-risk conditions. Finally, we analyzed the relationship of receipt of influenza vaccination with predictors for vaccination (age, educational level, marital status, employment, antenatal care, number of children, high-risk conditions, gestational age at birth, recommendation or offer of vaccination by health care provider, and knowledge and attitudes about vaccination) by bivariate and multivariate analysis (log-binomial regression). We present unadjusted and adjusted prevalence ratios (PRs) with 95% confidence intervals. Data were analyzed using STATA® software (version 14.0).

## Results

### Characteristics of study population

A total of 854 pregnant women were invited to participate in the survey and accepted by written consent to participate in the study (24 mothers refused to participate). Of those invited, 12 (1.4%) were excluded because they were not residents of Quito and only arrived at selected hospitals for delivery. Therefore, 842 pregnant women were included in the analysis.

The characteristics of the sample are described in Table 1. Almost three-quarters of participants in this survey were between 18 and 30 years old, 86% were mixed-race women and 58% finished high school. Most women were married or cohabited with a partner (79%), 44.2% were homemakers and approximately two thirds (65%) of women reported having at least one other child prior to this pregnancy. Nearly all women (98.7%) reported attending at least one antenatal visit and 81% reported more than four antenatal visits. Only 8% of women reported having chronic diseases.

**Table 1:** Characteristics of study population in Quito, Ecuador, 2016–2017 (*n* = 842)

Characteristics	n (%)
**Age**	
18–24	363 (43.1%)
25–30	260 (30.9%)
31–35	123 (14.6%)
≥ 36	96 (11.4)
**Race**	
White	27 (3.4%)
Mixed	723 (85.8%)
Indigenous	54 (6.4%)
Black	30 (3.5%)
Other	8 (0.9%)
**Education**	
Complete higher education or graduate degree	83 (9.9%)
Complete high school or incomplete higher education	407 (48.3%)
Complete basic education or incomplete high school	222 (26.4%)
Illiterate or incomplete basic education	130 (15.4%%)
**Marital status**	
Married	288 (34.2%)
Cohabited with a partner	376 (44.7%)
Separated / Widowed / Divorced - Never Married or Unmarried	178 (21.1%)
**Employment**	
Public or private employee	175 (20.8%)
Independent worker	172 (20.4%)
Homemaker	372 (44.2%)
Student	119 (14.1%)
Unemployed	4 (0.5%)
**Number of children (prior to this pregnancy)**	
0	295 (35.0%)
1–2	459 (54.5%)
3–6	88 (10.5%)
**Number of antenatal visits**	
0	11 (1.3%)
1 to 4	150 (17.8%)
≥ 5	681 (80.9%)
**Gestational age at birth**	
24–36 weeks	122 (14.5%)
37–42 weeks	720 (85.5%)
**High-risk conditions**	
No	775 (92.0%)
Yes	67 (8.0%)
**Received influenza vaccination (self-reported)**^**a**^	
Yes	308 (36.6%)
Confirmed with vaccination card	206 (66.9%)
No	534 (63.4%)
**Received influenza vaccination (vaccination card/medical records)**^**b**^	
Yes	206 (24.5%)
No	636 (75.5%)

^a^Vaccination reported by the women used for analysis

^b^Vaccination confirmed by vaccination card/medical records

### Vaccination rate in pregnant women

The percentage of women who reported having been vaccinated against influenza at any time in their pregnancy was 36.6%. Sixty percent of women have been vaccinated during the second trimester of pregnancy. Vaccination data was confirmed with the vaccination card and/or medical records in 67% of vaccinated women (Table 1).

### Knowledge and attitudes regarding influenza and influenza vaccination

**Fig. 1:**
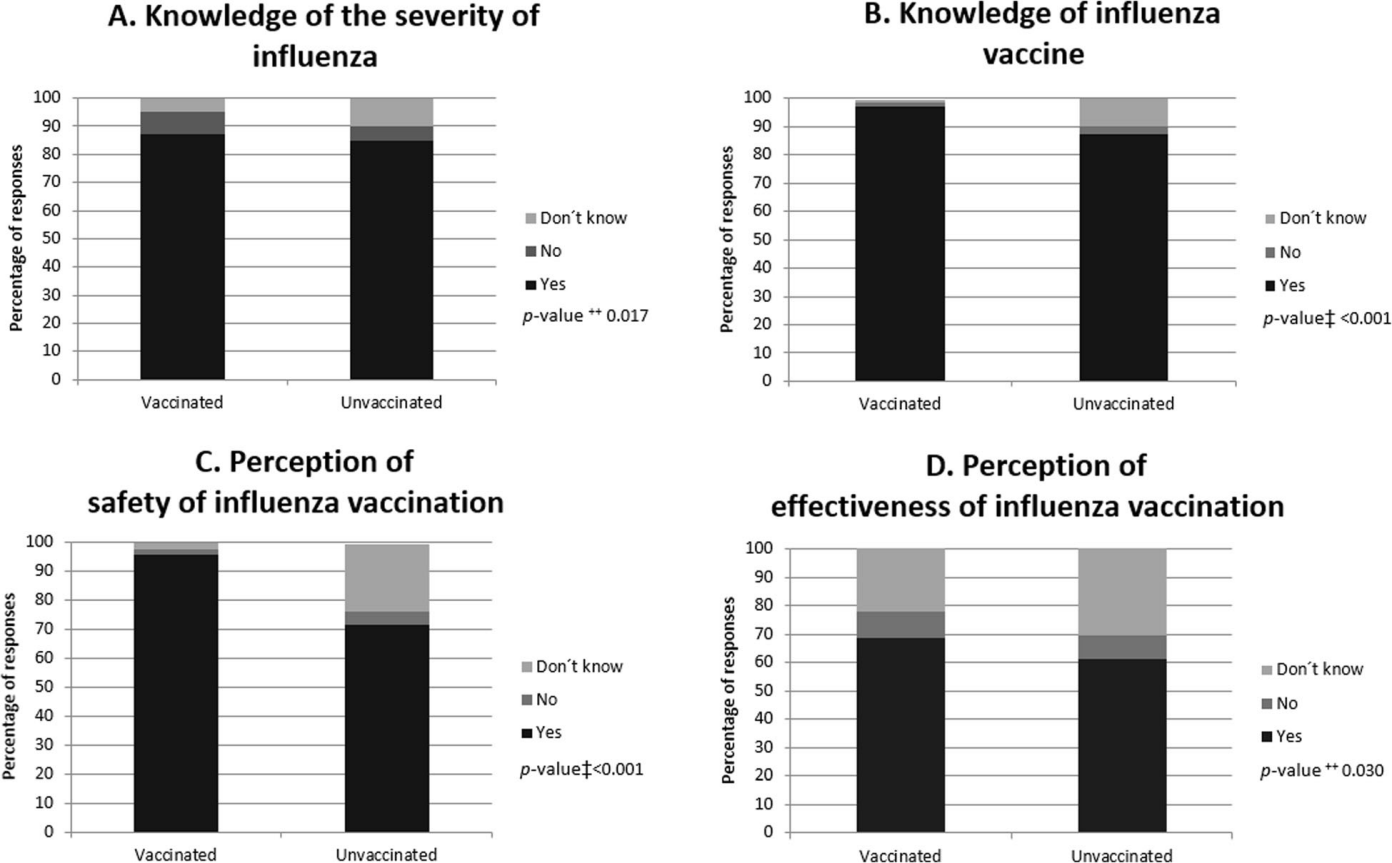
Knowledge and attitudes of women regarding influenza and influenza vaccination according to the vaccination status (*n* = 842), Quito-Ecuador, 2016–2017. Bars represent numbers in percentages. This figure refers to the following questions from the survey: **a** “Can influenza cause serious illness?”; **b** “There is a vaccine to prevent influenza?”; **c** “Are flu vaccines safe for me and my child during pregnancy?”; **d** “Can the flu vaccine protect against severe influenza?”. ^++^*X*^2^ test; ‡Fisher’s exact test

Knowledge about the severity of influenza and the existence of a vaccine was higher among women who reported having been vaccinated compared to those who reported not having been vaccinated (*p* = 0.017 and *p* < 0.001, respectively, Fig. 1a and b). Vaccinated women perceived that the influenza vaccine is safe (95.8% vs 71.7%, respectively) and effective (68.5% vs. 61.4%, respectively) in a higher proportion than unvaccinated women (*p* < 0.001 y *p* = 0.030, respectively, Fig. 1c and d).

### Reasons for not receiving influenza vaccination

The most frequent reason identified as a barrier to vaccination among pregnant women was the lack of recommendation/offer of the vaccine by the health provider (73.9%). Other reasons in smaller proportion were lack of access (9.0%), concern with the safety of the vaccine (6.2%), not wanting/needing the vaccine (3.7%) and other causes (7.3%) (Table 2). The most common reasons for non-vaccination among women with complete basic education or higher were also related to not having received a recommendation/offer of the vaccine by the health care provider, vaccine safety concerns and other reasons, whereas for women without any educational level or with incomplete basic education, not wanting/needing the vaccine and access barriers were the most common reason for non-vaccination. (*p* = 0.001, Table 2).

**Table 2:** Main reasons for not receiving the influenza vaccine during pregnancy (*n* = 520), Quito, Ecuador, 2016–2017

	Main reason						
	All n (%)	Concern about vaccine safety n (%)	Do not need/do not want n (%)	Access barriers^a^ n (%)	Did not receive recommendation/offer n (%)	Other reasons^**b**^ n (%)	***p***-value ǂ
**All**	520 (100)^c^	32 (6.2)	19 (3.7)	47 (9.0)	384 (73.9)	38 (7.3)	
**Age**							0.509
18–24	226 (43.5)	19 (59.4)	9 (47.4)	17 (36.2)	169 (44.0)	12 (31.6)	
25–30	159 (30.6)	8 (25.0)	4 (21.1)	18 (38.3)	117 (30.5)	12 (31.6)	
31–35	77 (14.8)	2 (6.2)	4 (21.0)	9 (19.1)	54 (14.1)	8 (21.0)	
≥ 36	58 (11.1)	3 (9.4)	2 (10.5)	3 (6.4)	44 (11.4)	6 (15.8)	
**Education**							0.001
Complete higher education or graduate degree	60 (11.5)	2 (6.3)	1 (5.3)	3 (6.4)	47 (12.2)	7 (18.4)	
Complete high school or incomplete higher education	244 (46.9)	16 (50.0)	6 (31.6)	21 (44.7)	180 (46.9)	21 (55.3)	
Complete basic education or incomplete high school	134 (25.8)	13 (40.6)	5 (26.3)	7 (14.9)	103 (26.8)	6 (15.8)	
Illiterate or incomplete basic education	82 (15.8)	1 (3.1)	7 (36.8)	16 (34.0)	54 (14.1)	4 (10.5)	
**Number of children (prior to this pregnancy)**							0.065
None	188 (36.2)	17 (53.1)	4 (26.3)	12 (25.5)	146 (38.0)	8 (21.0)	
1–2	285 (54.8)	12 (37.5)	12 (63.2)	29 (61.7)	208 (54.2)	24 (63.2)	
3–6	47 (9.0)	3 (9.4)	2 (10.5)	6 (12.8)	30 (7.8)	6 (15.8)	
**Number of antenatal visits**							0.621
≤ 4	117 (22.5)	7 (21.9)	6 (31.6)	13 (27.7)	85 (22.1)	6 (15.8)	
≥ 5	403 (77.5)	25 (78.1)	13 (68.4)	34 (72.3)	299 (78.9)	32 (84.2)	
**High-risk conditions**							0.615
No	472 (90.8)	28 (87.5)	19 (100)	42 (89.4)	349 (90.9)	37 (89.5)	
Yes	48 (9.2)	4 (12.5)	0 (0)	5 (10.6)	35 (9.1)	4 (10.5)	

This table refers to the following question from the survey: “of the reasons you listed, what is the main reason you will not get a flu vaccination this flu season?

^ǂ^ x^2^ test or Fisher’s test,

^a^Access barriers: “Vaccine unavailability (*n* = 23)”, “The health center is far from my home or opens at times that are not suitable for me (*n* = 11), “Sick when shot was available (*n* = 6)”, and other reasons related to access

^b^Most common other reasons were: “Don’t know”, “I had already been vaccinated before pregnancy”

^c^Fourteen people did not answer the question

### Provider recommendation and offer of influenza vaccination

**Fig. 2:**
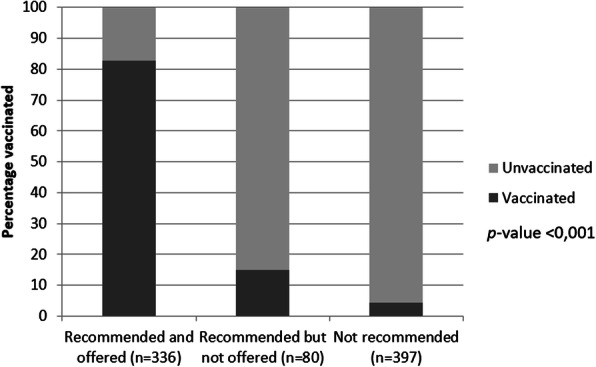
Vaccination against influenza during pregnancy according to the recommendation or offer of the vaccine by health personnel (*n* = 813). Quito-Ecuador, 2016–2017. Bars represent numbers in percentage

Among women who indicated that their health care provider recommended and offered the influenza vaccine, 82.7% reported having been vaccinated for influenza since the end of 2015. Among those who reported that their health care provider recommended but did not offer vaccination against influenza, 15.0% reported having been vaccinated for influenza. Finally, 4.3% of the respondents who did not receive either a recommendation or an influenza vaccination offer, reported having been vaccinated (*p* < 0.001, Fig. 2).

### Relationship between determinants and vaccination

A larger number of antenatal care visits, knowledge about vaccine safety, and having received recommendation (with or without offer of the vaccine) by health care personnel were associated with vaccination during pregnancy in both the bivariate and multivariate analysis (Table 3). Specifically, the vaccination rate was 1.67 times higher in women who reported having five or more antenatal controls during pregnancy than in women who reported having fewer than five controls and the association was maintained after adjustment by other predictors (adjusted PR 1.21, 95% CI 1.01–1.47). Women who perceived vaccination against influenza as safe had higher vaccination rates than those who did not (adjusted PR 1.53, 95% CI 1.03–2.37). Finally, women who reported receiving recommendation but were not offered vaccination and those who reported receiving both recommendation and were offered vaccination had 3.17 (95% CI 1.57–6.40) and 15.84 (95% CI 9.62–26.10) greater likelihood of having received the vaccine compared to women who did not receive a recommendation/offer.

**Table 3:** Determinants of influenza vaccination during pregnancy in Quito-Ecuador, 2016–2017

Variable	All ***n*** = 842	Vaccinated ***n*** = 308 n (%)	Crude PR CI 95%	Adjusted PR CI 95%
**Age**				
18–24	363	131 (36.1)	1.0	1.0
25–30	260	98 (37.7)	1.04 (0.85–1.29)	0.99 (0.86–1.15)
31–35	123	42 (34.2)	0.95 (0.71–1.25)	0.89 (0.72–1.11)
≥ 36	96	37 (38.5)	1.07 (0.80–1.42)	1.01 (0.84–1.22)
**Race**				
White	27	8 (29.6)	1.0	1.0
Afro-Ecuadorian	30	13 (43.3)	1.46 (0.72–2.98)	0.95 (0.68–1.33)
Mixed	723	270 (37.3)	1.26 (0.70–2.27)	0.87 (0.65–1.17)
Indigenous	54	15 (27.8)	0.94 (0.45–1.93)	0.74 (0.48–1.15)
Other	8	2 (25.0)	0.84 (0.22–3.20)	0.60 (0.22–1.64)
**Education**				
Complete higher education or graduate degree	83	23 (27.1)	1.0	1.0
Complete secondary education or incomplete higher education	407	157 (38.6)	1.39 (0.96–2.01)	1.27 (0.99–1.64)
Basic education complete or incomplete high school	222	82 (36.9)	1.33 (0.90–1.96)	1.30 (0.99–1.71)
Illiterate or incomplete basic education	130	46 (35.4)	1.28 (0.84–1.94)	1.39 (0.90–1.84)
**Marital status**				
Married	288	107 (37.2)	1.0	1.0
Cohabited with a partner	376	144 (38.3)	1.03 (0.84–1.26)	0.99 (0.87–1.13)
Separated / Widowed / Divorced - Never Married or Unmarried	178	57 (32.0)	0.86 (0.66–1.12)	0.95 (0.77–1.17)
**Employment**				
Housewife	172	58 (33.7)	1.0	1.0
Student	119	38 (31.9)	1.16 (0.91–1.47)	1.00 (0.86–1.17)
Unemployed	4	1 (25.0)	0.97 (0.72–1.29)	0.95 (0.77–1.17)
Public or private employee	175	61 (34.9)	0.92 (0.66–1.28)	0.88 (0.69–1.12)
Independent worker	372	150 (40.3)	0.72 (0.13–3.97)	0.82 (0.44–1.52)
**Number of children**				
0	88	37 (42.1)	1.0	1.0
1–2	459	169 (36.8)	1.06 (0.87–1.30)	0.90 (0.79–1.03)
3–6	295	102 (34.6)	1.22 (0.91–1.63)	1.03 (0.81–1.32)
**Antenatal Care**				
≤ 4	161	38 (23.6)	1.0	1.0
≥ 5	681	270 (39.7)	1.67 (1.25–2.25)^†^	1.21 (1.01–1.47)^†^
**Gestational age at birth**				
< 37 weeks	122	37 (30.3)	1.0	1.0
≥ 37 weeks	720	271 (37.6)	1.24 (0.93–1.65)	1.08 (0.91–1.28)
**High-risk conditions**				
No	775	290 (37.4)	1.0	1.0
Yes	67	18 (26.9)	0.72 (0.48–1.08)	0.91 (0.69–1.20)
**Distance to health center (minutes)**				
> 30 min	35	9 (25.7)	1.0	1.0
15–30 min	102	35 (34.3)	1.33 (0.71–2.49)	0.75 (0.54–1.05)
0–15 min	696	262 (37.6)	1.46 (0.83–2.59)	0.79 (0.57–1.08)
**Knowledge regarding influenza vaccine**				
No	54	25 46.3)	1.0	1.0
Yes	721	268 (37.2)	0.80 (0.59–1.09)	0.91 (0.73–1.12)
Do not know/no answer	67	15 (22.4)	0.48 (0.28–0.82)	0.90 (0.63–1.28)
**Knowledge about the transmission of the disease**				
No	87	34 (39.1)	1.0	1.0
Yes	666	251 (37.7)	0.96 (0.73–1.28)	1.10 (0.91–1.32)
Do not know/no answer	89	23 (25.8)	0.66 (0.43–1.03)	1.23 (0.93–1.63)
**Knowledge about the existence of vaccine**				
No	17	3 (17.7)	1.0	1.0
Yes	767	301 (39.2)	2.22 (0.79–6.24)	0.69 (0.25–1.91)
Do not know/no answer	58	4 (6.9)	0.39 (0.10–1.58)	0.55 (0.17–1.82)
**Perception about vaccine safety**				
No	32	6 (18.8)	1.0	1.0
Yes	678	295 (43.5)	2.32 (1.12–4.80)^†^	1.53 (1.03–2.37)^†^
Do not know/no answer	132	7 (5.3)	0.28 (0.10–0.78)	0.65 (0.33–1.28)
**Perception about vaccine effectiveness**				
No	72	29 (40.2)	1.0	1.0
Yes	539	211 (39.2)	0.97 (0.72–1.31)	0.87 (0.61–1.2)
Do not know/no answer	231	68 (29.4)	0.73 (0.52–1.03)	0.86 (0.68–1.09)
**Recommendation and offer of vaccine**				
No recommendation/non-offer	397	17 (4.3)	1.0	1.0
Recommendation / non-offer	80	12 (15.0)	3.50 (1.74–7.05)^†^	3.17 (1.57–6.40)^†^
Recommendation / offer	336	278 (82.7)	19.32 (12.1–30.85)^†^	15.84 (9.62–26.10)^†^

^†^*p*-value < 0.05

*CI* Confidence interval, *PR* Prevalence Ratio

## Discusion

Our study found a low influenza vaccination rate in pregnant women in Quito-Ecuador and identified some barriers that could contribute to low vaccination coverage. Those women who were vaccinated knew about the severity of influenza, about the existence of a vaccine, and perceived vaccination against influenza as safe and effective. The main barrier for not receiving the vaccine was the lack of recommendation/offer regarding influenza vaccine by health care providers. Among the determinants, recommendation/offer of vaccine increases the likelihood of vaccination in pregnancy. Other factors associated with vaccination were knowledge about vaccine safety and more than five antenatal care visits.

The vaccination rate reported in this study (36.6%) is lower than those reported for Ecuador in 2015 (55%) and 2016 (63%) and for those reported by other countries of the region, such as Bolivia (69%), Brazil (80%) and Argentina (100%) for 2016 [16]. However, the coverage could be underestimated due to the low percentage of women with perinatal or vaccination cards [28]. In Ecuador, the coverage of all vaccines (including the influenza vaccine) has shown a gradual decrease since 2013 to 2016, and a slight increase from 2017 [15] . The Evaluation of National Strategy of Immunizations revealed two elements related to this fact: 1) the Immunization Program underwent a transition, becoming part of the National Immunization Strategy. This fact implied a disaggregation of functions between different actors without an effective articulation of actions; and 2) the lack of budget allocation in a sustainable manner for operational activities of vaccination strategy [15]. Given these facts and the results of the present study, there is an urgent need to implement a contingency plan to improve short-term vaccination coverage and reduce the risk of transmission of vaccine-preventable diseases in Ecuador.

Our results are in agreement with previous studies that show that a compelling recommendation from a provider is one of the most important factors in a pregnant woman’s decision to get vaccinated [21, 25, 29–32]. Indeed, the lack of recommendation was a barrier for vaccination among pregnant women. Knowledge about influenza and vaccination by health workers has an impact on the decisions made regarding the vaccination of their patients and themselves. Studies show that maternal care providers with high levels of knowledge and positive attitudes consistently discuss and recommend influenza vaccine to their patients in greater proportion than other health providers [33–36]. Similarly, health professionals who know the national guidelines on influenza vaccination are more likely to discuss and recommend the vaccine than those who do not know them [37]. To our knowledge, there are no studies in Ecuador on the knowledge and attitudes of health workers regarding the influenza vaccination. Other studies demonstrate that health care workers are often reluctant to receive a vaccine [38, 39], have concerns about side effects, demonstrate a lack of faith in its efficacy and have concerns in the severity of the disease [40, 41]. Understanding health provider barriers is vitally important because it is not possible to overcome vaccination barriers among pregnant women if health providers themselves are not fully convinced about the benefits of maternal immunization.

Working to promote practices related to the recommendation and offer of influenza vaccination among antenatal care providers (physicians, obstetricians, nurses and midwifes) will be crucial to improving vaccination coverage during pregnancy. A recent study in five lower-middle and upper-middle income countries from Latin America reported a training gap related to maternal and neonatal immunization (MNI) programs in the pre-graduate curriculum of universities. Moreover, all five countries reported continuous training for health workers mostly addressed to personnel that administer vaccines, but not health care providers who recommend them [42]. Another study highlights the need to involve midwives and obstetricians in vaccine promotion and training as they usually have close and trust-based relationships with their pregnant patients [43]. Therefore, the incorporation or reinforcement of MNI programs in the university curriculum for the training of doctors, nurses, obstetricians, and midwives as well as continuous training for all antenatal care providers could be crucial for increasing influenza vaccine uptake during pregnancy.

In the present study, women who reported perceiving the influenza vaccine as safe and effective had the highest vaccination rates and vaccine safety concern was a reason for not receiving vaccination among 6.2% of non-vaccinated women. Lack of knowledge due to insufficient information about the safety of the influenza vaccine has previously been linked to lower vaccination rates [22, 23, 44]. The vaccine is considered safe throughout pregnancy and during lactation, and has been administered to pregnant women for many years without having observed adverse effects [1]. Therefore, efforts are needed to educate pregnant women and the population in general regarding the safety and effectiveness of the influenza vaccine to improve vaccination coverage of this risk group.

Having five or more antenatal visits increased the probability of vaccination which is in accordance to other studies [45–47]. Antenatal check-ups are essential to promoting the benefits of influenza vaccination and to offering the vaccine to pregnant women [48]. To increase vaccination coverage, it would be necessary to offer the influenza vaccine for a longer period rather than just one or two vaccination campaigns. This strategy would benefit women who have few prenatal visits or who are late in attending their first visit. Altogether, different strategies of vaccine delivery to pregnant women need to be evaluated to inform policy decisions in countries where influenza circulation is not confined to a single seasonal peak.

Our study showed different reasons for not being vaccinated according to educational level of women. The main reasons for lack of vaccination among illiterate women or with incomplete basic education was not need/want the vaccine and lack of access to vaccination. Studies have shown that people who have a higher education level and/or household income are more likely to receive preventive health services because they may have more knowledge about the importance of health-preventive care and the effectiveness of preventive strategies and more access to health-related services [49, 50].

Some limitations were identified in this study. Firstly, cross-sectional studies do not allow inferring causality because temporal sequence cannot be established. Second, the study sample was not randomly selected but rather a convenience sample, which makes generalization difficult and affects the external validity. We selected a population that is homogeneous with respect to socioeconomic level (lower and lower-middle class population) who use the public health system; therefore, the results could be representative of this group in Quito. Third, this study corresponds to the 2015–2016 influenza-vaccination campaign; however, the results are relevant considering the low vaccination coverage during the last years and the lack of information on the barriers related to vaccination during pregnancy in Ecuador. Finally, 30 % of vaccinated women lacked documentation of influenza vaccine status and self-report of vaccination could be affected by social desirability and forgetfulness; however, analysis of a subsample that included only those with written documentation of vaccination showed similar findings.

## Conclusion

In conclusion, the low rate of vaccination of pregnant women in Quito supports the need to establish health educational programs to increase the knowledge about seasonal influenza and on the efficacy and safety of vaccination among this population. These results also call for further studies on barriers of health providers regarding influenza vaccination in Ecuador. Education and training of all antenatal care providers, including obstetricians and midwives is needed to enhance their role as vaccinators, which could potentially increase the number of those willing to recommend and offer vaccination. In addition, the role of health workers in ensuring the success of home-based records (i.e. vaccination cards) of pregnant women should be emphasized to improve monitoring during pregnancy, childbirth, and postnatal period. Finally, other methods of vaccine delivery need to be evaluated, such as two-round antenatal care distribution or to incorporate influenza vaccination into other programs that focus on the most vulnerable pregnant women in tropical countries where influenza circulation is not confined to a single seasonal peak.

## Supplementary Information

**Additional file 1.** KAP questionnaire. English version of KAP questionnaire regarding influenza vaccination in pregnant women.

## Data Availability

The datasets used and/or analyzed during the current study are available from the corresponding author (Ana L. Moncayo) on reasonable request.

## Abbreviations

PR: Prevalence Ratio
95% CI: 95% confidence interval
MOPH: Ministry of Public Health
ALRI: Acute Lower Respiratory Infection
MNI: Maternal and Neonatal Immunization
KAP: Knowledge, Attitudes, and Practices

## References

[1] Somerville LK, Basile K, Dwyer DE, Kok J (2018). The impact of influenza virus infection in pregnancy. Future Microbiol.

[2] Goodnight WH, Soper DE (2005). Pneumonia in pregnancy. Crit Care Med.

[3] Jamieson DJ, Theiler RN, Rasmussen SA (2006). Emerging infections and pregnancy. Emerg Infect Dis.

[4] Wang X, Li Y, O'Brien KL, Madhi SA, Widdowson MA, Byass P (2020). Global burden of respiratory infections associated with seasonal influenza in children under 5 years in 2018: a systematic review and modelling study. Lancet Glob Health.

[5] Mina MJ, Klugman KP (2014). The role of influenza in the severity and transmission of respiratory bacterial disease. Lancet Respir Med.

[6] Organization WH (2012). Vaccines against influenza WHO position paper – November 2012. Wkly Epidemiol Rec.

[7] Katz MA, Gessner BD, Johnson J, Skidmore B, Knight M, Bhat N (2017). Incidence of influenza virus infection among pregnant women: a systematic review. BMC Pregnancy Childbirth.

[8] Vojtek I, Dieussaert I, Doherty TM, Franck V, Hanssens L, Miller J (2018). Maternal immunization: where are we now and how to move forward?. Ann Med.

[9] Thompson MG, Li DK, Shifflett P, Sokolow LZ, Ferber JR, Kurosky S (2014). Effectiveness of seasonal trivalent influenza vaccine for preventing influenza virus illness among pregnant women: a population-based case-control study during the 2010-2011 and 2011-2012 influenza seasons. Clin Infect Dis.

[10] Omer SB, Clark DR, Aqil AR, Tapia MD, Nunes MC, Kozuki N (2018). Maternal influenza immunization and prevention of severe clinical pneumonia in young infants: analysis of randomized controlled trials conducted in Nepal, Mali and South Africa. Pediatr Infect Dis J.

[11] Nunes MC, Cutland CL, Jones S, Downs S, Weinberg A, Ortiz JR (2017). Efficacy of maternal influenza vaccination against all-cause lower respiratory tract infection hospitalizations in young infants: results from a randomized controlled trial. Clin Infect Dis.

[12] Eick AA, Uyeki TM, Klimov A, Hall H, Reid R, Santosham M (2011). Maternal influenza vaccination and effect on influenza virus infection in young infants. Arch Pediatr Adolesc Med.

[13] Zaman K, Roy E, Arifeen SE, Rahman M, Raqib R, Wilson E (2008). Effectiveness of maternal influenza immunization in mothers and infants. N Engl J Med.

[14] Nunes MC, Cutland CL, Madhi SA (2018). Influenza vaccination during pregnancy and protection against pertussis. N Engl J Med.

[15] MSP (2017). Evaluación de la Estrategia Nacional de Inmunizaciones.

[16] PAHO/WHO (2016). Influenza Vaccine Coverage in countries and territories of the Americas.

[17] Yuen CYS, Tarrant M (2014). A comprehensive review of influenza and influenza vaccination during pregnancy. J Perinat Neonat Nurs.

[18] Arriola CS, Vasconez N, Thompson M, Mirza S, Moen AC, Bresee J (2016). Factors associated with a successful expansion of influenza vaccination among pregnant women in Nicaragua. Vaccine..

[19] Jung EJ, Noh JY, Choi WS, Seo YB, Lee J, Song JY (2016). Perceptions of influenza vaccination during pregnancy in Korean women of childbearing age. Hum Vaccin Immunother.

[20] Bartolo S, Deliege E, Mancel O, Dufour P, Vanderstichele S, Roumilhac M (2019). Determinants of influenza vaccination uptake in pregnancy: a large single-Centre cohort study. BMC Pregnancy Childbirth.

[21] Stark LM, Power ML, Turrentine M, Samelson R, Siddiqui MM, Paglia MJ (2016). Influenza vaccination among pregnant women: patient beliefs and medical provider practices. Infect Dis Obstet Gynecol.

[22] Vila-Candel R, Navarro-Illana P, Navarro-Illana E, Castro-Sánchez E, Duke K, Soriano-Vidal FJ (2016). Determinants of seasonal influenza vaccination in pregnant women in Valencia, Spain. BMC Public Health.

[23] Napolitano F, Napolitano P, Angelillo IF (2017). Seasonal influenza vaccination in pregnant women: knowledge, attitudes, and behaviors in Italy. BMC Infect Dis.

[24] Wilson RJ, Paterson P, Jarrett C, Larson HJ (2015). Understanding factors influencing vaccination acceptance during pregnancy globally: a literature review. Vaccine..

[25] Sheldenkar A, Lim F, Yung CF, Lwin MO (2019). Acceptance and uptake of influenza vaccines in Asia: a systematic review. Vaccine..

[26] WHO. Advocacy, communication and social mobilization for TB control: a guide to developing knowledge, attitude and practice surveys. Geneva: WHO; 2008.

[27] Instituto Nacional de Estadística y Censos I (2010). Fascículo provincial Pichincha.

[28] Dansereau E, Brown D, Stashko L, Danovaro-Holliday MC (2019). A systematic review of the agreement of recall, home-based records, facility records, BCG scar, and serology for ascertaining vaccination status in low and middle-income countries. Gates Open Res.

[29] Arriola CS, Mercado-Crespo MC, Rivera B, Serrano-Rodriguez R, Macklin N, Rivera A (2015). Reasons for low influenza vaccination coverage among adults in Puerto Rico, influenza season 2013-2014. Vaccine..

[30] Bödeker B, Walter D, Reiter S, Wichmann O (2014). Cross-sectional study on factors associated with influenza vaccine uptake and pertussis vaccination status among pregnant women in Germany. Vaccine..

[31] Blanchard-Rohner G, Meier S, Ryser J, Schaller D, Combescure C, Yudin MH (2012). Acceptability of maternal immunization against influenza: the critical role of obstetricians. J Matern Fetal Neonatal Med.

[32] Myers KL (2016). Predictors of maternal vaccination in the United States: an integrative review of the literature. Vaccine..

[33] Offeddu V, Tam CC, Yong TT, Tan LK, Thoon KC, Lee N (2019). Coverage and determinants of influenza vaccine among pregnant women: a cross-sectional study. BMC Public Health.

[34] Quattrocchi A, Mereckiene J, Fitzgerald M, Cotter S (2019). Determinants of influenza and pertussis vaccine uptake in pregnant women in Ireland: a cross-sectional survey in 2017/18 influenza season. Vaccine..

[35] Englund JA (2003). Maternal immunization with inactivated influenza vaccine: rationale and experience. Vaccine..

[36] Martinello RA, Jones L, Topal JE (2014). Correlation between healthcare workers’ knowledge of influenza vaccine and vaccine. Infect Control Hosp Epidemiol.

[37] Tong A, Biringer A, Ofner-Agostini M, Upshur R, McGeer A (2008). A cross-sectional study of maternity care Providers' and Women's knowledge, attitudes, and Behaviours towards influenza vaccination during pregnancy. J Obstet Gynaecol Can.

[38] Aguilar-Díaz FC, Jiménez-Corona ME (2011). Ponce-de-León-Rosales S. Influenza vaccine and healthcare workers. Arch Med Res.

[39] Seale H, Wang Q, Yang P, Dwyer DE, Wang X, Zhang Y (2010). Influenza vaccination amongst hospital health care workers in Beijing. Occup Med.

[40] Willis BC, Wortley P (2007). Nurses' attitudes and beliefs about influenza and the influenza vaccine: a summary of focus groups in Alabama and Michigan. Am J Infect Control.

[41] Dvalishvili M, Mesxishvili D, Butsashvili M, Kamkamidze G, McFarland D, Bednarczyk RA (2016). Knowledge, attitudes, and practices of healthcare providers in the country of Georgia regarding influenza vaccinations for pregnant women. Vaccine..

[42] Ropero Alvarez AM, Vilajeliu A, Magarinos M, Jauregui B, Guzman L, Whittembury A, et al. Enablers and barriers of maternal and neonatal immunization programs in Latin America. Vaccine. 2020. 10.1016/j.vaccine.2020.07.051. 10.1016/j.vaccine.2020.07.051 32943263

[43] Malik FA, Alonso JP, Sanclemente LN, Vilajeliu A, Gutierrez M, Gonzalez-Casanova I, et al. Health care providers perspectives about maternal immunization in Latin America. Vaccine. 2020. 10.1016/j.vaccine.2020.09.014. 10.1016/j.vaccine.2020.09.014 33127187

[44] Mayet AY, Al-Shaikh GK, Al-Mandeel HM, Alsaleh NA, Hamad AF (2017). Knowledge, attitudes, beliefs, and barriers associated with the uptake of influenza vaccine among pregnant women. Saudi Pharm J.

[45] Mendoza-Sassi RA, Cesar JA, Cagol JM, Duarte IA, Friedrich LM, Santos VKD (2015). 2010 a(H1N1) vaccination in pregnant women in Brazil: identifying coverage and associated factors. Cad Saude Publica.

[46] Blondel B, Mahjoub N, Drewniak N, Launay O, Goffinet F (2012). Failure of the vaccination campaign against a(H1N1) influenza in pregnant women in France: results from a national survey. Vaccine..

[47] Maher L, Dawson A, Wiley K, Hope K, Torvaldsen S, Lawrence G (2014). Influenza vaccination during pregnancy: a qualitative study of the knowledge, attitudes, beliefs, and practices of general practitioners in central and South-Western Sydney. BMC Fam Pract.

[48] Mak DB, Regan AK, Joyce S, Gibbs R, Effler PV (2015). Antenatal care provider's advice is the key determinant of influenza vaccination uptake in pregnant women. Aust N Z J Obstet Gynaecol.

[49] Christenson B, Lundbergh P (2002). Comparison between cohorts vaccinated and unvaccinated against influenza and pneumococcal infection. Epidemiol Infect.

[50] Worasathit R, Wattana W, Okanurak K, Songthap A, Dhitavat J, Pitisuttithum P (2015). Health education and factors influencing acceptance of and willingness to pay for influenza vaccination among older adults. BMC Geriatr.

