# Supplementary material for: Knowledge, attitudes and practices on influenza vaccination during pregnancy in Quito, Ecuador
Source: BMC Public Health. 2021 Jan 7;21:72. doi: 10.1186/s12889-020-10061-4 (PMC7791889; doi:10.1186/s12889-020-10061-4)
Supplement: Supplementary file 1 — Additional file 1. KAP questionnaire. English version of KAP questionnaire regarding influenza vaccination in pregnant women. [file 12889_2020_10061_MOESM1_ESM.docx]

**KAP QUESTIONNAIRE**

**1.- Code ____________________**

***For interviewer use only (Must be filled out before starting survey):***

1. **Informed consent was obtained:**

☐ Yes  ☐ No (If the answer is NO, finish the survey)

**Survey information**

1. **Survey date: _____/_____ / ________ (DD / MM / YY)**
2. **Interviewer ID: ____________________________________________________________**
3. **Survey location (Hospital): ___________________________________________________________**

**Respondent Information**

1. **What is your date of birth? ______/ _____/_______ (DD/MM/YY)**
2. **Where do you currently live (province/county/parish)?**

a. Province: _______________________ 88-☐ refused or don’t know

b. County: _______________________ 88-☐ refused or don’t know

c. Parish: _______________________ 88-☐ refused or don’t know

d. Neighborhood: _______________________ 88-☐ refused or don’t know

1. **What is your racial group background?**

1-☐ Afro-Ecuadorian   2-☐ White

3-☐ Mixed race 4-☐ Indigenous 5- Other, specify: ____________________________

1. **What is your highest level of education?**

1-☐ Illiterate 2-☐ Basic education completed  3-☐ Basic education incompleted

4-☐ Secondary school completed 5-☐ Secondary education incompleted

6-☐ University completed 7 -☐ University incompleted

8-☐ Post graduate degree completed 9-☐ Post graduate degree incompleted

1. **What is your marital status?**

1-☐ Married  2-☐ Cohabitating

3-☐ Separated / Widowed / Divorced 4-☐ Single (never married)

1. **Which of the following best describes your main work status before delivery?**

1-☐ Employee (public or private) with salary 2-☐ Independent worker

3-☐ Homemaker 4-☐ Student

5-☐ Unemployed (able to work) 6-☐ Unemployed (unable to work)

88-☐ Refuse

1. **Did you see anyone for antenatal care during the last pregnancy?**

1-☐ Yes  2-☐ No (**If the answer is NO, go to question No.16)**

1. **How many times did you receive antenatal care during the last pregnancy? ____________**

a-☐ Confirmed in the perinatal medical record (PMR)

1. **Date of the first prenatal visit _____/_____/_____ (DD/MM/YY)** a-☐ Confirmed in the PMR

99.  don’t know / There is no information in the PMR

1. **Gestational age at the time of the first antenatal visit _________ ☐ Weeks** a-☐ Confirmed in the PMR

99.  don’t know/ There is no information in the PMR

1. **Gestational age at the time of childbirth: __________☐ Weeks**
2. **What is your child’s date of birth? ____/____/_____ (DD/MM/YY)**

**18. What is the date of your last menstrual period (LMP)? ____/____/_____ (DD/MM/YY)** a-☐ Confirmed in the PMR

99.  don’t know/ There is no information in the PMR

**19. Do you have any sons or daughters to whom you have birth who are alive (not counting the one who has just been born)? ________ children**

**20. How old is son # 1 ?: _____**  years  months

**21 How old is son # 2 ?: _____**  years  months

**22. How old is son # 3 ?: _____**  years  months

**23. How old is son # 4 ?: _____**  years  months

**24. How old is son # 5 ?: _____**  years  months

**25. How old is son # 6 ?: _____**  years  months

**26. How old is son # 7 ?: _____**  years  months

**27. Have you ever been vaccinated against influenza in previous pregnancies?**

1.  Yes; 2.  No; 99.  don’t know/ no answer; N A-  Not apply (first child, or first living child)

**28. Have you ever been diagnosed by your health care provider with any of the following conditions?**

| **Disease** | **1.Yes** | **2.No** | **99. Do not know** |  | **Disease** | **1. yes** | **2.No** | **99. Do not know** |
| --- | --- | --- | --- | --- | --- | --- | --- | --- |
| 1. Chronic heart disease (read heart failure, high blood pressure, arrhythmias) |  |  |  |  | f. Mellitus diabetes |  |  |  |
| 1. Asthma |  |  |  |  | g. Chronic kidney disease |  |  |  |
| 1. Bronchitis |  |  |  |  | h . Immunosuppression (read HIV/AIDS – Low immune system, problems with the immune system) |  |  |  |
| 1. COPD (read Chronic obstructive pulmonary disease) |  |  |  |  | 1. Cancer |  |  |  |
| 1. Cystic fibrosis |  |  |  |  | j. Cerebrovascular accident (read cerebral ischemia or hemorrhage) |  |  |  |

**A. Knowledge regarding influenza vaccination**

1. **Please tell me if you agree or disagree with the following statements regarding influenza vaccination:**
   1. **Influenza can cause severe illness.**

1. Yes; 2.  No; 99.  don’t know / no answer

- 1. **Influenza can be spread from person to person.**

1. Yes; 2.  No; 99.  don’t know / no answer

- 1. **There is a vaccine to prevent influenza.**

1. Yes; 2.  No; 99.  don’t know / no answer

- 1. **Influenza vaccine is safe for me and my child during pregnancy.**

1. Yes; 2.  No; 99.  don’t know / no answer

- 1. **Influenza vaccine can protect against severe influenza.**

1. Yes; 2.  No; 99.  don’t know / no answer

**B. Attitudes regarding influenza vaccination**

1. **Have you been recommended influenza vaccine by any health care worker in your last pregnancy?**

1. Yes; 2.  No; 99.  don’t know / no answer

1. **Have you been offered influenza vaccine by any health care worker in your last pregnancy?**

1. Yes; 2.  No; 99.  don’t know / no answer

1. **Have you taken up seasonal influenza vaccine during your last pregnancy?**

1. Yes; 2.  No; 99.  don’t know / no answer

**(If the answer is NO or DO NOT KNOW / NO ANSWER, go to question 38)**

**If the answer is YES, go to question 33:**

1. **Where did you taken up influenza vaccine?**

1. Public health facility 2.  Private health facility

3.  Other, specify _________________ 99. don’t know / no answer

**34. What made you decide to get vaccinated (tick all that apply)?**

1. To protect me against influenza

2. To protect my baby against influenza

3. My healthcare provider recommended me

4 My healthcare provider offered me

5. Other, specify ________________________________________________________________________________________________________

99. Don’t know / no answer

1. **We need to verify the vaccination date.**

**a. Did you bring your immunization card with you?** 1. Yes; 2. No

**b. If you don't bring with you, Could I call you in a few days so that you can read me the vaccination date?**

1. Yes; 2. No; 99. don’t know / no answer

**What is your phone number? Phone______________________________ Cell phone ___________________________**

**When should I call you? Date: ___/___/_____, Hour: __________**

**c. Could you provide us with your address if we are unable to reach you by phone?**

1. Yes; 2. No; 99. don’t know / no answer

**d. Address:**

____________________________________________________________________________________________________

__________________________________

**36.** **Influenza vaccination date: _____/_____/______ (DD/MM/YY)**

Vaccination could not be verified.

**37. Source of information:**

1. Immunization card 2.  Perinatal Medical Record (PMR) 3.  Phone call

**(Go to question No.40)**

**C. Practices regarding influenza vaccination**

**38. If you were not vaccinated against influenza in the last pregnancy, what were the reasons? (tick all that apply)**

- 1. **You don´t like to get vaccinated** 1. Yes; 2. No; 99. don’t know / no answer
  2. **You are afraid of needles** 1. Yes; 2. No; 99. don’t know / no answer
  3. **You get sick when you get vaccinated** 1. Yes; 2. No; 99. don’t know / no answer
  4. **You are afraid that the vaccine could harm your baby** 1. Yes; 2. No; 99. don’t know / no answer
  5. **You think the vaccine could harm you** 1. Yes; 2. No; 99. don’t know / no answer
  6. **The vaccine was not recommended to you during** 1. Yes; 2. No; 99. don’t know / no answer

**pregnancy**

- 1. **You were not offered the vaccine during pregnancy** 1. Yes; 2. No; 99. don’t know / no answer
  2. **The vaccine is too expensive** 1. Yes; 2. No; 99. don’t know / no answer
  3. **You don’t know where to get vaccinated** 1. Yes; 2. No; 99. don’t know / no answer
  4. **The health center is too far** 1. Yes; 2. No; 99. don’t know / no answer
  5. **It is very dangerous for you to go to the**

**vaccination center** 1. Yes; 2. No; 99. don’t know / no answer

- 1. **The health center opens at times that are not**

**convenient for you** 1. Yes; 2. No; 99. don’t know / no answer

- 1. **Other__________________________________________________________________________________**
  2. **None of the above**

**39. Of the reasons that you mentioned, which was the most important? (fill in this question only if people indicated several reasons for not getting vaccinated)**

**____________________________________________________________________________________________________________**

**____________________________________________________________________________________________________________**

**40. How far is the vaccination center from your home?**

a. Walking: _______minutes

b. Driving: _______minutes

1. **What do you think is the best way to inform pregnant women about the influenza vaccination?**

1-☐  Direct communication from health personnel to pregnant mothers.

2-☐  Availability of audiovisual information in the health facility.

3-☐ Dissemination of information through mass communication media (radio, television).

4-☐  Dissemination of information by political, religious and community organizations.

5-☐  Other (specify):______________________________________________________________________

99-☐  Don’t know / no answer

**THANK YOU FOR PARTICIPATING**!
